# An improved method for in vitro feeding of adult female *Dermanyssus gallinae* (poultry red mite) using Baudruche membrane (goldbeater’s skin)

**DOI:** 10.1186/s13071-020-04471-x

**Published:** 2020-11-19

**Authors:** Francesca Nunn, Jessica Baganz, Kathryn Bartley, Sarah Hall, Stewart Burgess, Alasdair J. Nisbet

**Affiliations:** 1 Moredun Research Institute, Edinburgh, UK; 2 Scottish Rural Agricultural College, Edinburgh, UK

**Keywords:** Baudruche membrane, *Dermanyssus gallinae*, In vitro feeding, Goldbeater’s skin, Poultry red mite

## Abstract

**Background:**

*Dermanyssus gallinae*, or poultry red mite (PRM), is an important ectoparasite in laying hen, having a significant effect on animal welfare and potentially causing economic loss. Testing novel control compounds typically involves in vitro methodologies before in vivo assessments. Historically, in vitro methods have involved PRM feeding on hen blood through a membrane. The use of hen blood requires multiple procedures (bleeds) to provide sufficient material, and the use of a larger species (e.g. goose) could serve as a refinement in the use of animals in research.

**Methods:**

The in vitro feeding device used was that which currently employs a Parafilm™ M membrane (Bartley et al.: Int J Parasitol. 45:819–830, 2015). Adult female PMR were used to investigate any differences in mite feeding, egg laying and mortality when fed goose or hen blood. Effects on these parameters when PRM were fed through either the Parafilm™ M membrane or the Baudruche membrane alone or through a combination of the membrane with an overlaid polyester mesh were tested using goose blood.

**Results:**

Poultry red mites fed equally well on goose or hen blood through the Parafilm™ M membrane, and there were no significant differences in mortality of PRM fed with either blood type. A significant increase (*t* test: *t* = 3.467,* df* = 4, *P* = 0.03) in the number of eggs laid per fed mite was observed when goose blood was used. A 70% increase in PRM feeding was observed when the mites were fed on goose blood through a Baudruche membrane compared to when they were fed goose blood through the Parafilm™ M membrane. The addition of an overlaid polyester mesh did not improve feeding rates. A significant increase (analysis of variance: *F*_(3, 20)_ = 3.193, *P* = 0.04) in PRM egg laying was observed in mites fed on goose blood through the Baudruche membrane compared to those fed goose blood through the Parafilm™ M membrane. A mean of 1.22 (standard error of the mean ± 0.04) eggs per fed mite was obtained using the Baudruche feeding device compared to only 0.87 (SEM ± 0.3) eggs per fed mite using the Parafilm™ M device when neither was combined with a polyester mesh overlay.

**Conclusion:**

The in vitro feeding of adult female PRM can be readily facilitated through the use of goose blood in feeding devices with the Baudruche membrane. 
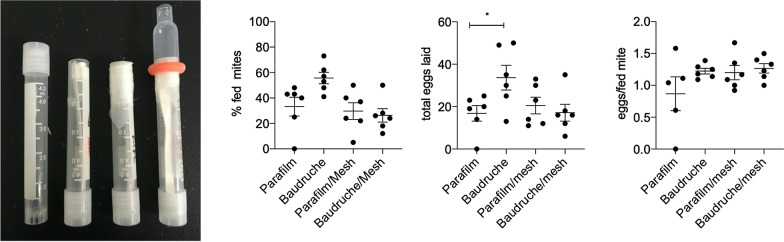

## Background

Infestation of laying hen houses with the poultry red mite (PRM), *Dermanyssus gallinae*, is a major animal welfare and economic problem for the egg-producing industry worldwide, costing the poultry industry in excess of €231 million per year in the EU alone [1]. Current treatment options are limited and often ineffective. PRM infestations cause serious welfare and production issues in the birds as a result of their nocturnal blood-feeding activities and potential as competent vectors for bacterial and viral pathogens [1–3]. Novel methods of controlling PRM (novel biopesticides and plant-derived products; semiochemicals and growth-regulators; vaccines etc.) are typically tested initially using small numbers of PRM in in vitro efficacy assays, followed by field testing using large numbers of both parasites and hens [4–6]. The in vitro feeding devices employed in these assays contain the mites, allowing them to feed on hen blood through a membrane, such as day-old chick skin [7, 8], or artificial membranes, such as the Parafilm™ M membrane [9, 10]. This methodology has technical limitations, not least due to the requirement for high numbers of replicates to overcome variable feeding rates when using Parafilm™ M and issues around supply, uniformity and quality when using chick skin [7, 8]. In addition, to supply sufficient hen blood for these assays, multiple invasive procedures are required involving venepuncture on hens’ wing veins. The aim of this study was to investigate the use of goose blood (which can be obtained in far higher volumes per procedure than hen blood) as a higher welfare alternative food source for the assays and goldbeater’s skin (also known as Baudruche membrane) as an alternative feeding membrane that may enable improved feeding of adult female PRM.

## Methods

### Blood samples

Chicken and goose whole blood samples were collected by wing vein venepuncture into a 1.5-ml capacity Eppendorf tube or syringe containing heparin, respectively (Sigma-Aldrich Co. Ltd., Dorset, UK), to give a final heparin concentration of 20 units/ml blood. Hen blood from four hens was pooled for further use; individual goose bleeds were used for each in vitro experiment. Trials were carried out on the day of blood collection, and blood was kept at room temperature (RT) until needed. The hens used were pullets aged approximately 20 weeks, housed in groups in floor pens. Male geese approximately 12 months old were used and housed in predator-proofed, enriched paddocks.

### Parasite material

Poultry red mites were collected and stored as previously described [11]. Briefly, *D. gallinae* of mixed stage and sex were collected at 3-weekly intervals from a commercial egg-laying unit. Prior to the feeding assays, mites were stored in vented 75-cm^2^ canted tissue culture flasks (Corning Inc., Corning, NY, USA) at RT for 7 days to allow blood digestion to occur, after which they were stored at 4 ℃ for up to 3 weeks.

### In vitro feeding device

**Fig. 1:**
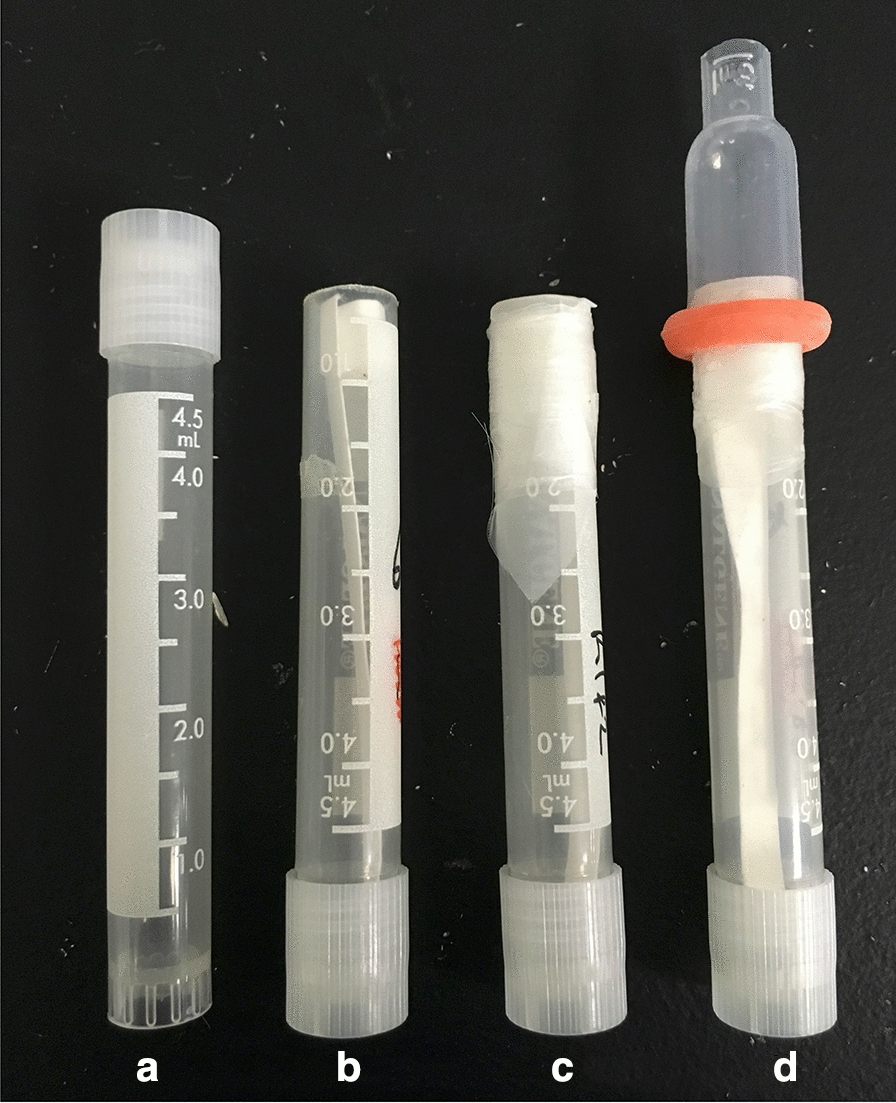
Sequence of construction of in vitro feeding device for poultry red mites. The closed end of a 5-ml cryotube (**a**) is cut off, the tube is inverted and a piece of filter paper inserted (**b**). Mites are added to the tube, and a square of mesh, either Baudruche membrane or Parafilm™ M, is placed over the cut-off end and held in place by a strip of Parafilm™ M stretched around the outside of the tube (**c**). A cut-down pastette bulb is then placed over the membrane, and a watertight seal is obtained by using a castration ring (**d**). Blood is placed inside the pastette bulb, and the device is placed in an incubator in the dark to allow mite feeding to commence

Feeding devices (Fig. 1) were constructed as described previously [3, 9, 12]. For the initial experiments comparing hen and goose blood as food sources, the feeding membrane used was Parafilm™ M. For the experiments comparing feeding membranes, the membranes were either a 2 × 2-cm square of Parafilm™ M membrane or Baudruche membrane (Preservation Equipment Ltd, Diss, UK), with the latter held in place by wrapping a strip of Parafilm™ M over the edges of the Baudruche membrane, around the outside of the tube, before the modified pipette bulb goose blood reservoir was placed over the top (Fig. 1). In the experiments to assess the impact of an overlaid polyester mesh on the feeding membranes, to improve mite attachment, a polyester mesh (Plastok Ltd, Birkenhead, UK) with an aperture width of 105 µm and a depth of 63 µm [10] was overlaid on the ventral aspect of the membranes used in the devices and heparinised goose blood was used as the food source.

### Feeding assays

Mites removed from storage at 4 ℃ were maintained at RT for 20 min to allow motile mites to migrate to the cap of the flask. Only motile, adult female mites were collected from the flask caps and transferred to the feeding devices (50 mites per device), following which the devices were sealed. Once all feeding devices contained mites, heparinised blood was added to the devices, which were then incubated in the dark in an incubator (model MLR-351H; Sanyo, Osaka, Japan) at 39 ℃ and 85% relative humidity (RH) for 3 h. Following incubation, mites were recovered from the devices, and each fed mite was transferred into a single-well of a 96-well tissue culture plate (Costar, Corning, NY, USA) which was then sealed using AeraSeal™ tape (Sigma-Aldrich Co.). Plates were placed into an incubator at 25 ℃ and 85% RH, and the mites observed using a stereo microscope at 3, 24, 48 and 144 h to record mite egg laying and mortality.

For the comparison of adult female PRM feeding rates, egg laying and mortality when fed goose blood or hen blood through a Parafilm™ M membrane, we performed a single experiment with three replicate assays per food source. For the comparison of adult female PRM feeding rates, egg laying and mortality when the PRM were fed goose blood through either a Parafilm™ M or a Baudruche membrane (or each of these membranes overlaid with the polyester mesh), we performed two repetitions of the experiment with three replicate assays per device in each.

### Statistical analysis

Unpaired Student’s* t* tests were performed on mite feeding, mortality and fecundity data for the comparison of mites fed on goose or hen’ blood. An analysis of variance (ANOVA) was used to determine variance between the two experiments, which were held on different days, to compare the different membranes. ANOVA using Dunnett’s multiple comparisons was used to compare any differences between the Parafilm™ M membrane and Parafilm™ M membrane + mesh, Baudruche membrane and Baudruche + mesh. To examine any difference between the Parafilm™ M membrane and Parafilm™ M membrane + mesh and Baudruche membrane and Baudruche + mesh, we used an ANOVA using Sidak’s multiple comparisons test. All analyses were carried out using GraphPad Prism v8 (GraphPad Software, San Diego, CA USA; www.graphpad.com).

## Results

### Comparison of feeding between goose and hen blood

Goose and hen blood were equally successful in terms of the numbers of PRM feeding on each blood source and survival of the mites following feeding (Table 1). There was a 26% increase in total egg production in mites fed on goose blood compared to those fed on hen blood, and the number of eggs laid per fed mite was significantly higher in those mites fed on goose blood (*t* test: *t* = 3.467,* df* = 4, *P* = 0.03).

**Table 1:** Poultry red mite feeding, egg laying and cumulative mortality 144 h after the mites had been fed with hen or goose blood

Test parameter	hen’ blood	Goose blood	*P*^a^
Number of mites feeding	19.0 ± 3.5	19.0 ± 5.5	0.96
Total number of eggs	28.6 ± 7.7	35.3 ± 9.4	0.61
Eggs laid per fed mite	1.56± 0.08	1.87 ± 0.03	0.03
Number of dead adult mites after 144 h	1.00 ± 0.6	1.00 ± 0.6	≥ 0.99

Mean numbers of mites and eggs are shown (*n* = 3) with standard error of the mean (SEM)

^a^Unpaired* t* test

### Comparison of PRM feeding on goose blood through Parafilm™ M, Baudruche membranes and in those membranes overlaid with polyester mesh

**Fig. 2:**
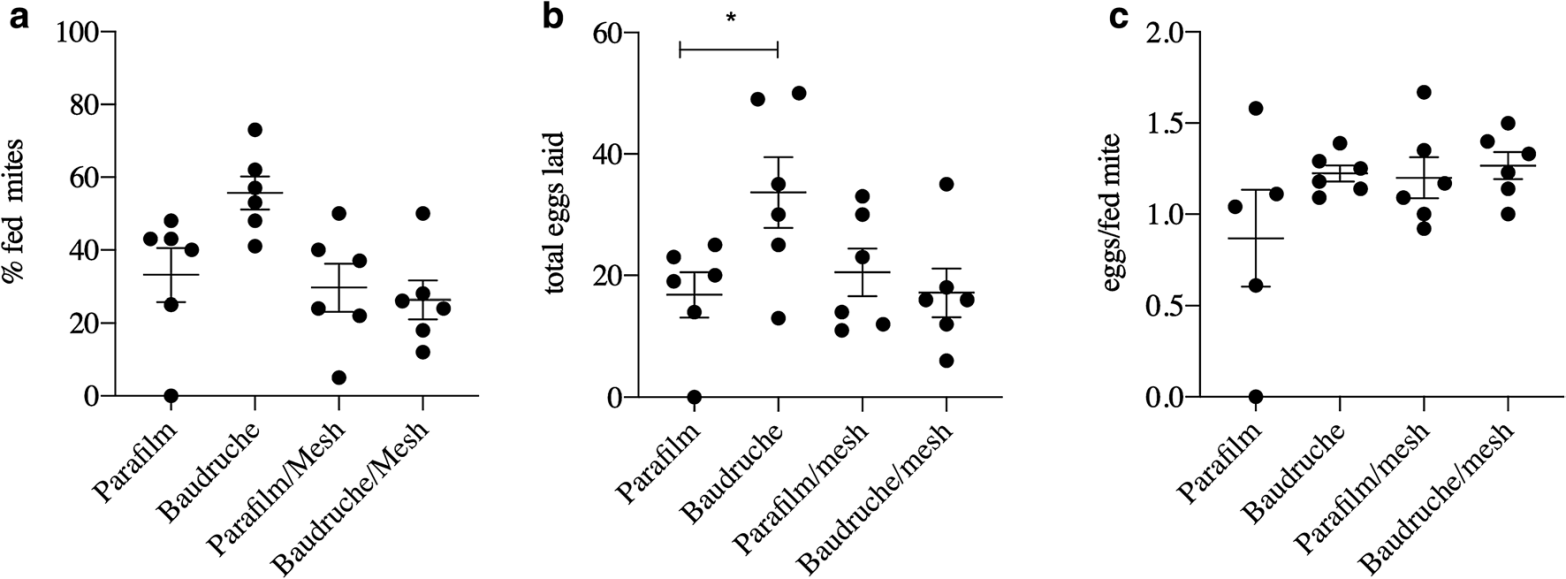
Feeding and egg laying of poultry red mites fed on goose blood through different membranes.** a** Feeding success rates of mites through different membranes,** b** total eggs laid over the course of the experiment,** c** eggs laid per fed mite when fed on goose blood through different membranes. Bars: Mean + standard error of the mean (*n* = 6). A significant increase in the number of eggs laid by mites feeding on goose blood through the Baudruche membrane without an overlaid polyester mesh was demonstrated (indicated with an asterisk on** b** (analysis of variance: *F*_(3, 20)_ = 3.193, *P* = 0.04)

No statistically significant differences were demonstrated within the same treatment groups in the two repetitions of the experiment in terms of feeding, egg laying, progeny per fed mite or mortality; consequently, replicates from the two repetitions of the experiment were combined for further analysis. Comparison of feeding rates, fecundity and mortality between PRM fed with goose blood through the different membranes is shown in Table 2. The Parafilm™ M membrane in one of the feeding devices developed a split during the incubation period, which is common with this type of membrane, whereas none of the Baudruche membranes failed. No feeding occurred in the failed device, and so data from this replicate were not included in the analysis. A 70% increase in the mean number of PRM feeding was observed in mites fed on goose blood through a Baudruche membrane compared to a Parafilm™ M membrane (Fig. 2; Table 2); however, due to the high levels of variability in the feeding levels of mites feeding through a Parafilm™ M membrane, this increase was not statistically significant (*P* = 0.1). Addition of an overlaid polyester mesh did not improve feeding rates on either membrane; rather, a significant (ANOVA: *F*_(3,20)_ = 3.1, *P* = 0.04) decrease in feeding was noted (Table 2) in the devices containing Baudruche + mesh compared to the Baudruche membrane alone. Mites which had fed through Baudruche membrane (without overlaid mesh) produced double the number of eggs of those fed through a Parafilm™ M membrane (ANOVA: *F*_(3, 20)_ =  3.193, *P* = 0.04) over the course of the experiment (Table 2; Fig. 2).

**Table 2:** Comparison between numbers of adult female poultry red mites feeding, egg laying, eggs laid per fed mite and mortality using goose blood as a food source and either Parafilm™ M, Baudruche membrane or a combination of either with an overlaid polyester mesh

Test parameter	Parafilm M membrane^a^	Baudruche membrane	Parafilm M membrane + mesh	Baudruche membrane + mesh
Number of mites feeding	16.0 ± 3.6	25.8 ± 4.2	17.5 ± 3.9	13.2 ± 2.7
Total number of eggs laid	16.8 ± 3.7	33.7 ± 5.8	20.5 ± 3.9	17.1 ± 3.8
Eggs/ fed mite	0.87 ± 0.3	1.22 ± 0.04	1.21 ± 0.1	1.27 ± 0.07
Number of dead adult mites after 144 h	0.4 ± 0.24	1.3 ± 0.6	0.7 ± 0.21	0.3 ± 0.21

Mean values are shown with SEM. *n* = 6 except where stated otherwise

^a^*n* = 5 due to failure of one device, which led to zero feeding

## Discussion

We have demonstrated that goose blood is an excellent food source for laboratory-maintained PRMs and that the Baudruche membrane is a superior feeding membrane compared to the traditionally used Parafilm^TM^ M membrane for in vitro feeding assays. Geese are well suited as blood donor animals as their size means they can donate more blood per procedure than hen (approx. 20 fold). Generally, blood collected from individual hen is pooled for use in feeding assays, and the amount collected can be limiting in terms of number of replicates and/or size of experiment. A statistically significant increase in eggs laid per fed mite was demonstrated using goose blood compared to hen blood in the initial experiment using the Parafilm^TM^ M-only device.

To our knowledge, this is the first published report on the use of Baudruche membrane in the feeding of *D. gallinae*, although this membrane has been used for tick feeding in conjunction with silicone as recently as 2019 [13] and for mosquitoes as early as 1964 [14]. Chick skin has been used successfully to allow in vitro feeding of PRM [4, 7, 8], but the availability and ease of use of artificial membranes has led to more frequent use of the latter. Membranes such as Parafilm™ M [9] or Nescofilm coated with a hen skin extract [10] are used and can work well. However, Parafilm™ M membranes often fail due to the need to stretch the membrane to make it sufficiently thin for mites to be able to feed successfully, and Nescofilm is no longer commercially available. Fed mites are difficult to recover from a device with membrane failure, possibly leading to misleading mortality data [5]. In addition, the rate of device failure and highly variable feeding rates require additional replicates, with the associated increased invasive sampling of hens. Therefore, a more reliable in vitro feeding device increases hen welfare in terms of refinement in number of procedures required for successful data collection.

Krull et al. 2017 [15] suggested that thin membranes for the feeding of tick larvae and nymphs could be obtained using Baudruche membrane coated in silicone or even by using Baudruche membrane alone. As previous attempts at using silicone with lens tissue, as described by Kröber and Guerin [16], did not result in PRM feeding in our laboratory (data not shown), we hoped that the reported high tensile strength of the Baudruche membrane alone would suffice and that it would be thin enough to allow the mites to feed without damaging the membrane. The addition of a mesh support has been successfully used in previous tick studies [14, 16, 17], resulting in enhanced attachment times of ticks to membranes. We therefore decided to test a mesh that had already demonstrated utility in the feeding of PRM [11] to determine if attachment was enhanced. No difference was observed in the feeding rates or egg laying with the Parafilm^TM^ M membrane-only system and the Parafilm^TM^ M + mesh system. A significant decrease in feeding was demonstrated between the Baudruche membrane + mesh system and the Baudruche membrane alone, possibly due to a lack of tension in the Baudruche membrane in the latter system and the difficulty faced by mites trying to attach through both the mesh and membrane compared to membrane alone. Greater variability was observed in both feeding rates and in number of eggs laid per fed mite when using only Parafilm^TM^ M when compared to Parafilm^TM^ M + mesh, whereas neither feeding rates or eggs laid per mite were affected by the addition of the mesh. In addition, no membrane failures were observed when using the Parafilm^TM^ M with the overlaid mesh.

We observed in this study that mites that had fed on goose blood through the Baudruche membrane were fully engorged when compared to those that had fed through the Parafilm™ M membrane. This repletion level may explain the differences in the trend of increased egg production in the mites fed in the Baudruche membrane system. Therefore, the textured surface of the Baudruche membrane may facilitate easier mite attachment, enabling them to feed to repletion, when compared to the smooth Parafilm™ M membrane. The robust performance, availability and good feeding rates indicates that the Baudruche membrane is a useful alternative to chick skin and artificial membranes for use in PRM feeding.

## Conclusion

When applied to novel interventions being delivered to PRMs via hen blood (e.g. novel vaccines and systemic acaricides), in vitro studies have previously suffered from highly variable mite feeding rates and high background mortality of mites when using the in vitro feeding system [4, 5]. A more reliable in vitro feeding device reduces the necessity of higher replicates and therefore the volume of blood required, leading to a refinement in animal procedures.

A more reliable in vitro feeding method is ideal for initial screening of new control compounds and for those studies that do not require on-hen testing (e.g. RNAi studies). We have demonstrated the potential of using Baudruche membrane for in vitro feeding of adult PRM, with further studies planned to examine its use for the other hematophagous life stages of the parasite.

## Data Availability

The datasets supporting the conclusions of this article are provided within the article and can be acquired from the corresponding author on request.
